# Argatroban for an alternative anticoagulant in HIT during ECMO

**DOI:** 10.1186/s40560-017-0235-y

**Published:** 2017-06-28

**Authors:** Alain Rougé, Felix Pelen, Michel Durand, Carole Schwebel

**Affiliations:** 1 Service de Réanimation Médicale, CHU des Alpes, CS 10217, 38043 Grenoble Cedex 9, France; 2 Service de Réanimation cardio-vasculaire et thoracique, CHU des Alpes, CS 10217, 38043 Grenoble Cedex 9, France; 3 Université de Grenoble Alpes France, Grenoble, France; 4 Inserm U1039, Radiopharmaceutiques biocliniques, Domaine de la Merci, 38700 la Tronche, France

**Keywords:** Argatroban, Extracorporeal membrane oxygenation, Heparin-induced thrombocytopenia

## Abstract

**Background:**

Extracorporeal membrane oxygenation (ECMO) have become more frequently used in daily ICU practice, heparin-induced thrombocytopenia (HIT) is a rare but life-threatening complication while on extracorporeal membrane oxygenation (ECMO). HIT confirmation directly impacts on anticoagulant strategy requiring no delay unfractionated heparin discontinuation to be replaced by alternative systemic anticoagulant treatment.

**Case presentation:**

We report two clinical cases of HIT occurring during ECMO in various settings with subsequent recovery with argatroban and provide literature review to help physicians treat HIT during ECMO in clinical daily practice.

**Conclusions:**

HIT during ECMO is uncommon, and despite the absence of recommendation, argatroban seems to be an appropriate and safe therapeutic option. Finally, there are not enough arguments favouring routine circuit change in the event of HIT during ECMO

## Background

Extracorporeal membrane oxygenation (ECMO) have become more frequently used in daily ICU practice in various clinical settings either in adult or paediatric population [1]. During ECMO, anticoagulant therapy is mandatory with current recommendations based on unfractionated heparin (UFH) [1]. However, systemic anticoagulation achieved by heparin administration exposes patients to the risk of heparin-induced thrombocytopenia (HIT) [2]. Type II HIT is a rare entity, with an incidence of 5% when using UFH ranging from 1 to 10% while on ECMO. Underlying physiopathology involves immunoglobulin G targeting multimolecular complexes of platelet factor 4 (PF4) and polyanion heparin [3], leading to potential thromboembolic life-threatening complications. Related mortality varies from 10 to 30% [3, 4], and diagnosis may be more difficult under ECMO because of frequent concomitant-associated thrombocytopenia [5]. We describe two cases of HIT occurring during ECMO and discuss the management.

## Case Presentation

### Case report 1

A 49-year-old Caucasian man was referred to the emergency department in December 2014 for gradually worsening dyspnoea and haemoptysis. The patient was obviously an active smoker and heavy drinker and was socially isolated. At admission, the patient exhibited a respiratory rate at 17/min with peripheral arterial oxygen saturation at 94% in room air, no haemodynamic compromise (BP 145/120 mmHg, HR 157/min) and central temperature at 37.2 °C. ECG showed a sinus tachycardia at 120 bpm, without conduction or ST abnormalities. Platelet count was 202 G/L. The thoracic computed tomographic angiography exhibited bilateral subsegmental pulmonary embolism with multiple pulmonary infarcts. Ultrasonography of the lower limbs was normal. Treatment including enoxaparin, amoxicillin and clavulanic acid was initiated, and he was transferred to the medical ward. Recurrence of haemoptysis with persistent tachycardia led to his prompt transfer to the ICU on the next day. Transthoracic echocardiography demonstrated severe cardiac dysfunction with left ventricular ejection fraction (LVEF) of 15%, left ventricle dilation (left ventricle end-diastolic diameter at 59 mm), no right ventricle enlargement, aortic velocity time integral (VTI) at 5 cm and systolic pulmonary hypertension (PAPs 30 mmHg). Because of circulatory failure and hyperlactatemia related to obvious cardiogenic shock, the patient was given dobutamine and noradrenaline and was intubated and mechanically ventilated. Coronary angiography resulted normal; infectious screening and immunological tests for thrombophilia were negative. Additional tests reported various abnormalities with low plasma selenium at 0.77 μmol/L (Nl 0.81-1.38), zinc 10.6 μmol/L (Nl 12.4-18.3) and vitamin PP (B36) 32 μmol/L (Nl38-58). Alcohol-induced dilated cardiomyopathy decompensated by subsegmental pulmonary embolism was the final clinical diagnosis. Despite the pharmacological treatment optimization, the patient remained in poor clinical and haemodynamic conditions leading to right femoral-femoral veno-arterial ECMO implementation on day 2.

**Fig. 1:**
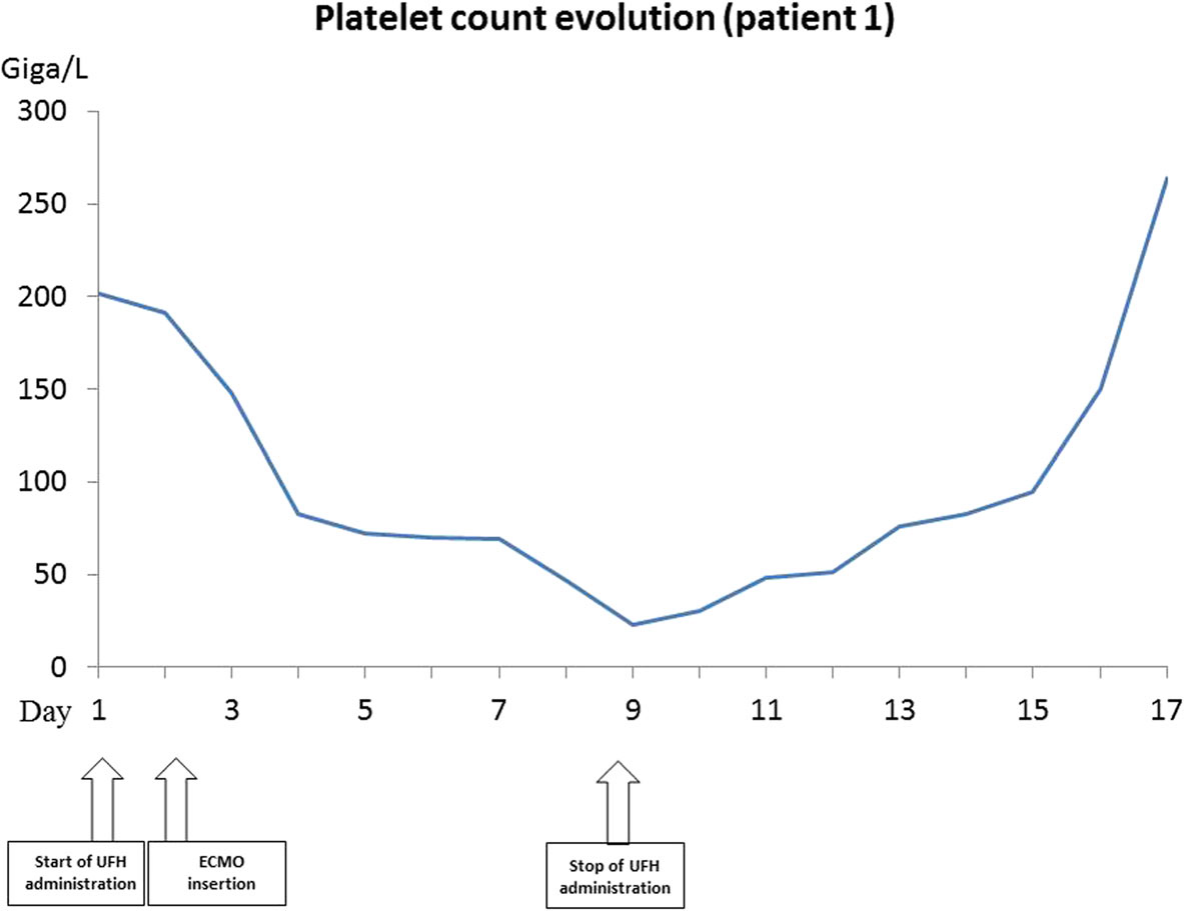
Platelet count evolution of patient 1

Clinical course was marked by transient atrial fibrillation and thrombopenia reported to 23 G/L on day 8 versus 83 G/L on day 3 (see Fig. 1), leading to platelet transfusion. The level of anti-PF4 antibodies measured by ELISA (IgG) was 0.775 (positivity threshold value at 0.146). Platelet aggregation test was subsequently positive on day 10. The diagnosis of type 2 HIT was established, and parenteral anticoagulant treatment required for on-going ECMO replaced by argatroban at the initial dose of 0.2 μg/kg/min. Change of extracorporeal circuit (catheters, membrane and lines) was not required. Biological and clinical outcome was good with platelet recovery being effective on day 6 after UFH removal and weaning from ECMO initiated on day 8 to be completed on day 12 after admission in ICU. The patient was extubated on day 20. Complementary myocardial MRI confirmed dilated left ventricular cardiomyopathy with dilatation of the left atrium and a medial strip of fibrosis. Transition treatment with danaparoid followed by vitamin K antagonist was provided so that the patient could have been transferred to the cardiology ward before rehabilitation. He finally was discharged to home 2 months after initial admission. Six-month follow-up with alcohol withdrawal and angiotensin-converting-enzyme inhibitor and beta-blocker treatment demonstrated a 50% LVEF.

### Case report 2

A 69-year-old patient was admitted to the emergency department for dyspnea. He suffered from advanced COPD (Gold III) and complicated diabetes with peripheral arteriopathy with right superficial femoral artery stenosis. He described dyspnea since 3 months and orthopnea for 1 week. The transthoracic echocardiography revealed a 3-cm posterior pericardial effusion with a right opacity on the chest X-ray with haemodynamic compromise. The effusion was surgically drained. The patient was intubated because of hypoxemia and developed a multiple organ failure requiring continuous haemodialysis. The aetiology was a pneumococcal pneumonia treated with amoxicillin. The patient developed then ARDS due to extensive bilateral pneumonia associated with a right-lung abscess. Because of a poor oxygenation despite the protective ventilation and NO administration, a veno-venous jugulo-femoral ECMO was inserted percutaneously on day 9. The procedure was complicated by a cardiac arrest due to hyperkaliemia, hypercapnia and respiratory acidosis promptly managed with IV epinephrine (6 mg) and haemodialysis via the ECMO circuit. The patient became haemodynamically unstable requiring high doses of vasopressors. Initial blood tests at ECMO initiation revealed a normal platelet count.

**Fig. 2:**
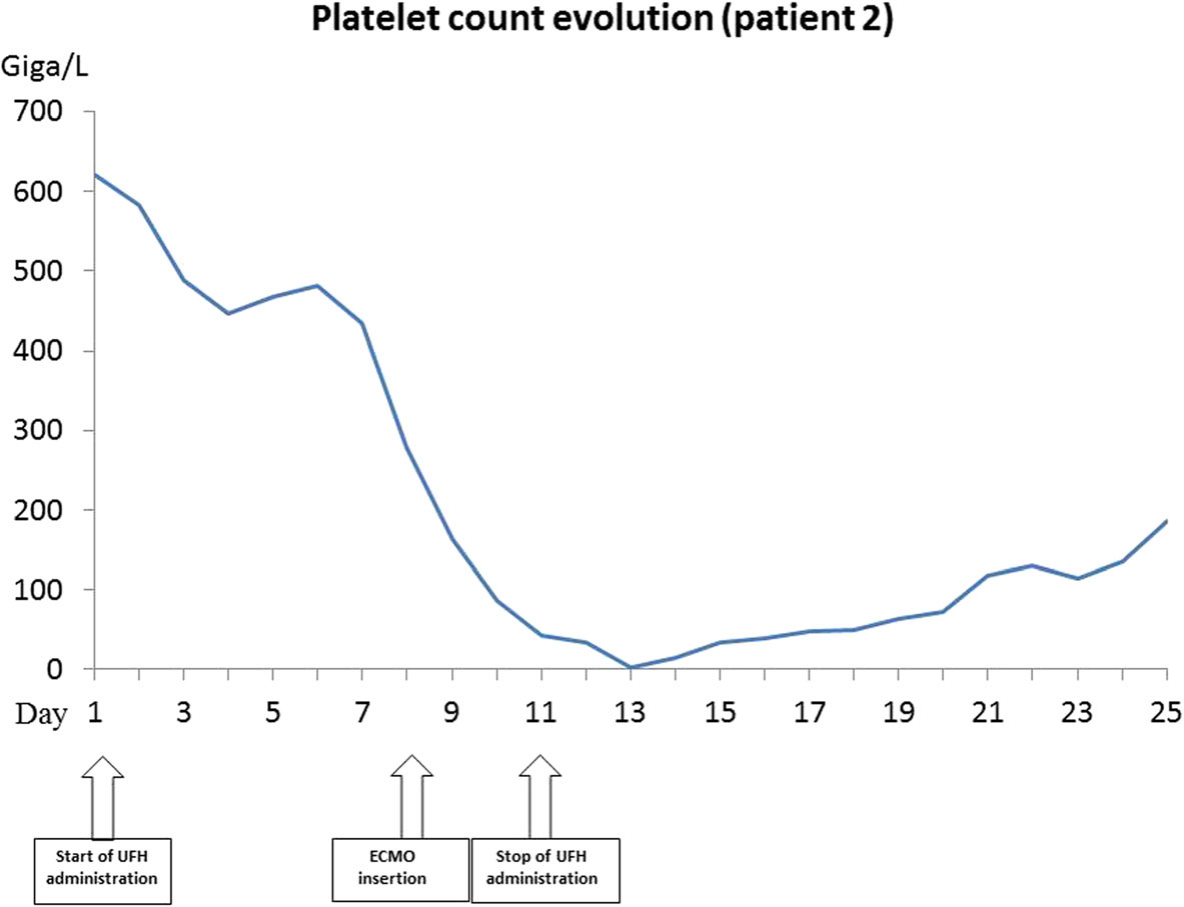
Platelet count evolution of patient 2

While on ECMO, he demonstrated a daily significant drop in platelet count from day 7 reaching a nadir at 3 G/L on day 12. The diagnosis of a HIT was confirmed through quantitative immuno-assay and platelet aggregation test on days 13 and 16, respectively. The UFH infusion was stopped on day 12 and replaced by argatroban. The ECMO circuit needed to be changed on day 12 because of a clot on the membrane (see Fig. 2). Argatroban was introduced at 1 μg/kg/min, which was lowered to 0.5 μg/kg/min because of a liver failure and a PT at 30%. The ECMO membrane was replaced on day 15. On day 20, the platelet count was normal and the ECMO was successfully removed. The patient gradually improved on all plans but sharply deteriorated again on day 30 while he was still treated by argatroban with efficient anticoagulation. Body scanner imaging showed an ischemic right colitis related to recent poor haemodynamic conditions without evidence of mesenteric thrombosis. The patient underwent a colectomy. In the postoperative course, the patient developed multi-organ failure with metabolic acidosis and hyperlactatemia leading to death.

## Discussion

Extracorporeal membrane oxygenation (ECMO) is a procedure providing circulatory and respiratory assistance, used for decades in cardiac surgery and postoperative course being nowadays more routinely used in non-specialised ICU [1]. Veno-venous ECMO (VV-ECMO) has become a therapeutic option for severe respiratory failure ARDS [1] and veno-arterial ECMO (VA-ECMO) for refractory cardiogenic shock. Acute and end-stage cardiac failure may be eligible indications for ECMO either as a bridge to recovery or transplantation [6]. Unfractionated heparin remains the “recommended” anticoagulant strategy during ECMO despite the potential risk of HIT. We reported two HIT during ECMO successfully managed by argatroban as a safe substitution therapy leading to platelet count recovery.

HIT is a rare entity especially in an ECMO situation. Diagnosis may be challenging under ECMO because of frequent associated thrombocytopenia from various origins (multi-organ failure, sepsis, platelet activation and consumption, bleeding, dilution). This thrombocytopenia may be linked to the non-biological surface causing an inflammatory response. Two types of HIT are described: type I thrombocytopenia as benign non-immune origin of early occurrence without thrombotic complications, and spontaneously resolving in spite of continued treatment with heparin; type II thrombocytopenia, potentially devastating, of immune origin and late onset. The term heparin-induced thrombocytopenia (HIT) refers to type II thrombocytopenia. HIT is a clinico-biological syndrome induced by antibodies, often IgG isotype, which mostly recognise platelet factor 4 (PF4) modified by heparin. Resulting platelet activation and coagulation activation explain subsequent venous and/or arterial thrombosis. Thrombocytopenia with >50% fall of platelets’ count between the fifth and tenth day following administration of heparin favours type II TIH diagnosis. In our cases, the diagnosis is made if a platelet aggregation test is positive, this being more specific than an antiPF4 antibody assay [3]. Anticoagulant treatment is necessary in an ECMO situation and as treatment for HIT in view of the risk of thrombosis. Apart from peripheral thrombotic risk increased by the presence of cannulae, one of the major issues is the risk of thrombosis from the ECC circuit [7].

HIT treatment consists in stopping the heparin as soon as possible [2], administering a non-heparinic anticoagulant and avoiding platelet transfusion. Alternative anticoagulant treatments include direct thrombin inhibitors: argatroban, bivalirudin or lepirudin, and *danaparoid sodium* (anti-Xa factor heparinoid) [8].

Respected to its pharmacological properties, i.e. short half-life, low rate of renal elimination [9] with predominant hepatic elimination conjugated with an easy to use manipulation, effective monitoring availability, argatroban appears suitable for HIT with ECMO. It has been considered as a safe therapeutic option in HIT patients at high hemorrhagic risk and with renal failure, particularly in an ICU setting [10]. Severe hepatic dysfunction (Child-Pugh Class C) is regarded a contraindication for argatroban administration as accumulation occurs. Argatroban is a synthetic molecule derived from l-arginine which anticoagulant effect is concentration-dependent targeting the active site of thrombin. It is metabolised by the liver into inactive nontoxic metabolites and is excreted through the faeces, independently of temperature. Its low distribution volume of 180 ml/kg is increased by extracorporeal circuit, and therefore does not diffuse in the tissues but in the extracellular space. Administration regimens have been detailed previously. Loading dose should be avoided, and starting dose on ECMO has to be cautious from 0.2 μg/kg/min [10] to 2 μg/kg/min according to targeted aPTT (activated partial thromboplastin time).

Coagulation monitoring during Argatroban is the most important point. Argatroban effects are currently monitored by aPTT (activated partial thromboplastin time) with a target range from 1.5 to 3.0 times the baseline values. Closed aPTT monitoring is expected to be checked 2 h after starting infusion to avoid excessive anticoagulation and bleeding complications. The dose will be increased by 0.05 μg/kg/min to obtain the appropriate target value, and the aPTT should be performed 2 h after the initiation of infusion and after every dosage adjustment until the steady-state aPTT is 1.5–3.0 times the initial baseline value and cannot surpass 100 s [11–13]. Nevertheless, the aPTT tends to show a ceiling effect, and the ecarin clotting time (ECT) or the ecarin chromogenic assay (ECA-T) may be considered preferable tests in patients who require very high-dose treatment [14]. Finally, similarly to the other thrombin inhibitors, argatroban does not have a specific antidote in case of severe bleeding.

Bivalirubin is also a first-line treatment in the event of HIT linked to cardiac surgery [12]. Its use is considered to be effective and safe because of its short half-life (25 min) and its elimination mostly via the thrombin cleavage enzyme [12]. The selected dosage for ECMO application is a continuous infusion from 0.5 to 2.5 mg/kg/h more or less, preceded by a bolus of 0.5 mg/kg, with a targeted ACT of between 300 and 350 s or a target ECT of between 400 and 550 s [15]. Danaparoid sodium is an anti-Xa factor heparinoid with a long half-life (25 h). This treatment, used in an ECMO situation [15] at a dose of between 200 and 300 U/h is monitored via the anti-Xa activity which can be difficult (anti-Xa activity targets between 0.6 and 0.8 U/ml) [15] and is no more available [16]. Lepirubin was the first direct thrombin inhibitor, with a half-life of 80 min and renal elimination. It was used as an alternative in the event of HIT during cardiac surgery, but carries a risk of renal failure and anaphylactic shock [15] and is no more available. Finally, a case of HIT treatment with fondaparinux has also been described, but since this treatment has neither antidote nor biological monitoring, it seems difficult to generalise its use.

Guidelines targeting ECMO circuit change in the event of HIT are lacking. Maintaining heparin-coated circuit despite the UFH treatment removal may result in persistent thrombocytopenia [17]. However, it is most likely that heparin which is chemically bound cannot diffuse into the blood and perform a conformational change necessary to become a target for PF4 antibodies and to bind with PF4 to platelets. Koster et al. compared ECMO circuit with heparin-coated and non-coated circuits and did not found any enhancement of heparin-PF4-IgG complex-associated immunologic or thrombogenic reactions with heparin-coated system [18]. Time course evolution of platelet count in both cases without systematic change of ECMO circuit at time of TIH diagnosis excludes active heparin interaction and immunologic effect. Ranucci et al. found no effect of biocompatible circuits on death after cardiac surgery [19]. To date, there are no enough arguments favouring routine circuit change in the event of HIT and most of the cases reported showed good evolution without changing the circuit [19–22].

## Conclusion

HIT during ECMO is uncommon. Functional platelet aggregation test remains accurate and relevant as the diagnostic tool in this clinical setting. Specific therapeutic management is based on stopping heparin to move to an alternative anticoagulant treatment with immediate action. Argatroban seems to be an appropriate and safe therapeutic option. However further studies are required to confirm efficacy and safety profile respected to other available molecules. No recommendation on ECMO circuits changing can be made to date.

## Abbreviations

ECMO: Extracorporeal membrane oxygenation
HIT: Heparin-induced thrombocytopenia
LVEF: Left ventricular ejection fraction
UFH: Unfractioned heparin
